# Titanium Implant Osseointegration Problems with Alternate Solutions Using Epoxy/Carbon-Fiber-Reinforced Composite

**DOI:** 10.3390/met4040549

**Published:** 2014-12-05

**Authors:** Richard C. Petersen

**Affiliations:** Restorative Sciences, Biomaterials and Biomedical Engineering, University of Alabama at Birmingham, SDB 539, 1919 7th Avenue South, Birmingham, AL 35294, USA; richbme@uab.edu; Tel.: +1-205-934-6898

**Keywords:** titanium, composite, bisphenol polymer, carbon fiber, osseointegration, corrosion, infection, estrogen, microbiocircuit, semiconductor

## Abstract

The aim of the article is to present recent developments in material research with bisphenyl-polymer/carbon-fiber-reinforced composite that have produced highly influential results toward improving upon current titanium bone implant clinical osseointegration success. Titanium is now the standard intra-oral tooth root/bone implant material with biocompatible interface relationships that confer potential osseointegration. Titanium produces a TiO_2_ oxide surface layer reactively that can provide chemical bonding through various electron interactions as a possible explanation for biocompatibility. Nevertheless, titanium alloy implants produce corrosion particles and fail by mechanisms generally related to surface interaction on bone to promote an inflammation with fibrous aseptic loosening or infection that can require implant removal. Further, lowered oxygen concentrations from poor vasculature at a foreign metal surface interface promote a build-up of host-cell-related electrons as free radicals and proton acid that can encourage infection and inflammation to greatly influence implant failure. To provide improved osseointegration many different coating processes and alternate polymer matrix composite (PMC) solutions have been considered that supply new designing potential to possibly overcome problems with titanium bone implants. Now for important consideration, PMCs have decisive biofunctional fabrication possibilities while maintaining mechanical properties from addition of high-strengthening varied fiber-reinforcement and complex fillers/additives to include hydroxyapatite or antimicrobial incorporation through thermoset polymers that cure at low temperatures. Topics/issues reviewed in this manuscript include titanium corrosion, implant infection, coatings and the new epoxy/carbon-fiber implant results discussing osseointegration with biocompatibility related to nonpolar molecular attractions with secondary bonding, carbon fiber *in vivo* properties, electrical semiconductors, stress transfer, additives with low thermal PMC processing and new coating possibilities.

## 1. Introduction

Titanium alloys developed in the 1940s for aircraft were made available to orthopedic surgeons as biomaterials for bone implants approximately at the same time [1] and were also tested earlier with cat femurs during the late 1930s [2]. Since World War II, the two dominant titanium alloys have been 98%–99.6% commercially pure titanium (CPTi) and titanium with 6% aluminum and 4% vanadium (Ti-6Al-4V) alloy [1-4]. CPTi has four grades of oxygen content from 0.18%–0.40% that increase the yield strength with variable other small amounts of metal impurities [1-3]. CPTi is generally reserved for dental applications due to an extremely stable oxide TiO_2_ thin surface layer that resists corrosion under physiologic conditions [1-3] and forms a fine interfacial direct metal to bone contact as osseointegration [1-4]. Titanium metal has a relatively low modulus for metal [1-3,5]. Subsequent low modulus materials close to bone then reduce problems with “stress shielding” so that more uniform stress transfer occurs between the implant and bone to prevent bone resorption from periods with lack of pressure [1,5]. Ti-6Al-4V has been used for dental implants and although stronger than CPTi, biocompatibility is a concern from aluminum and vanadium ions released [3]. Ti-6Al-4V has also been used for orthopedic hip implant stems, but the Ti-alloy is particularly prone to geometrical notch sensitivity with crack propagation and further wears excessively as the chief concern [1]. Titanium alloys are also used to repair craniofacial defects caused by trauma, surgical removal of cysts and tumors, infections, fractures that do not join and congenital or developmental conditions [4]. However, titanium failures occur and appear related to factors that discourage stabilized bone osseointegration such as trauma from overloading, micromotion and surgical burden [6] to support inflammation without proper healing and in a small percentage infection next to exposed metal surface as the final destructive mechanisms for implant loosening [4,7]. Also, the healing response involves serum protein adhesion to the implant that can promote bacterial attachment to a biomaterial surface [7].

Recent technology moreover supported through aerospace/aeronautical development with epoxy/carbon-fiber-reinforced composites has demonstrated far-reaching osseointegration increases when compared to Ti-6Al-4V alloy in animal research [5]. The bisphenol epoxy backbone structure was developed early in 1936 as the first synthetic estrogen [8] where estrogen influences are known to produce anabolic stimulating bone formation and osteoblast differentiation [5,9-12]. Further, fiber-reinforced composite can offer superior mechanical properties than metals on a strength-to-weight basis for both strength and modulus [5,13,14]. Occlusal forces interact with titanium implants more harshly than natural tooth structure because of intimate bone osseointegration contact without a damping protective periodontal ligament [15,16] where titanium metal cannot adsorb damaging energy similar to a polymer matrix composite (PMC) [17]. In fact, *in vivo* animal testing with extreme loads produced defects lateral to osseointegration between bone and metal implant [16,18]. Conversely, in relation to encouraging test results [5] PMCs with carbon fiber reinforcement can supply densities/modulus much closer to bone [1,2,5] than titanium [5,14] for improved mechanical deformation providing viscoelastic damping energy adsorption/dissipation [2,5,17] and healthy stress transfer with tissues/cell membranes [5]. Also, carbon-fiber-reinforced PMC has electrical conductivity/resistivity properties bordering similarly on bone properties with polymer insulated carbon-fiber conductive biocircuits [5,19] to support biocompatible physiological relationships [5]. In addition, thermoset polymer matrix and carbon fiber both offer covalent bonding opportunity to give strong bone structure support with excellent osseointegration [5]. Further, epoxy/carbon-fiber-reinforced PMC does not corrode to release Lewis acid-stimulating metal particles that can initiate an inflammatory response with aseptic bone implant loosening [5]. Finally, low-thermal polymer-based thermoset processing allows incorporation of minerals and even low-temperature organic additives for major tissue design-engineering [5].

## 2. Corrosion

Corrosion is a diffusion interfacial electron-transfer process that occurs on the surface of metals. Titanium reacts with oxygen electrochemically rapidly in the presence of water to form a fine oxide layer of TiO_2_ that prevents further oxidation [3,20], Equation (1). The TiO_2_ surface layer protects titanium under normal biologic conditions to regenerate if removed by reactive corrosion equilibrium products as passivation barrier formation and confers high corrosion resistance [2,3,21]. Titanium can form an oxide layer 10 angstroms thick in a millisecond and 100 angstroms in a minute [3,22]. In the passivated state, TiO_2_ biomaterials generally corrode less than 20 μm/year [22]. TiO_2_ as Ti^4+^ and O^2−^ with even numbers as the most common oxidation states [23] are considered to provide molecular interaction similarities to bone [21] possibly by coordination as simple ionic bonds with analogous even oxidation states through calcium phosphate mineral, Ca_3_(PO_4_)_2_, from divalent Ca^2+^ and O^2−^ [23].

(1) $$\mathrm{Ti}+O_{2}=\mathrm{Ti}O_{2}$$

Still, all metal implants are not perfectly passive in a hostile corrosive biological environment to have some solubility and are subject to metal dissolution with the formation of metal cations (M^+^) and electrons (e^−^), Equation (2) [1,3,21]. Aqueous concentrations of dissolved molecular oxygen in the tissue react and remove electrons to form hydroxyl anion [1,3,21], Equation (3), that helps drive corrosion through Equation (2) [3]. Further, metal cations are removed to polarize water forming a Lewis acid, Equation (4) [21,23,24] that can then accelerate corrosion through Equation (2). Also, with low pH, normal biologic extracellular chlorine can form hydrochloric acid [21] that may attack titanium [20,22,25] with undesirable bone responses [22]

(2) $$M=M^{+}+e^{-}$$

(3) $$O_{2}+2H_{2}O+4e^{-}=4OH^{-}$$

(4) $$M^{+}+H_{2}O=(M^{+})(HO^{-}-H^{+})=M^{+}(OH^{-})+H^{+}$$

Capillary distance is a measure of lower oxygen concentration or increased acid and lower pH where zero O_2_ concentrations develop at about a 0.2 mm tissue space [26-28]. Resulting lower oxygen concentrations near the implant surface without an oxygenated blood supply are unable to satisfy intracellular mitochondrial requirements during energy synthesis to form water [29,30], Equations (5) and (6).

(5) $$O_{2}+2e^{-}+2H^{+}=H_{2}O_{2}$$

(6) $$H_{2}O_{2}+2e^{-}+2H^{+}=2H_{2}O$$

**Figure 1:**
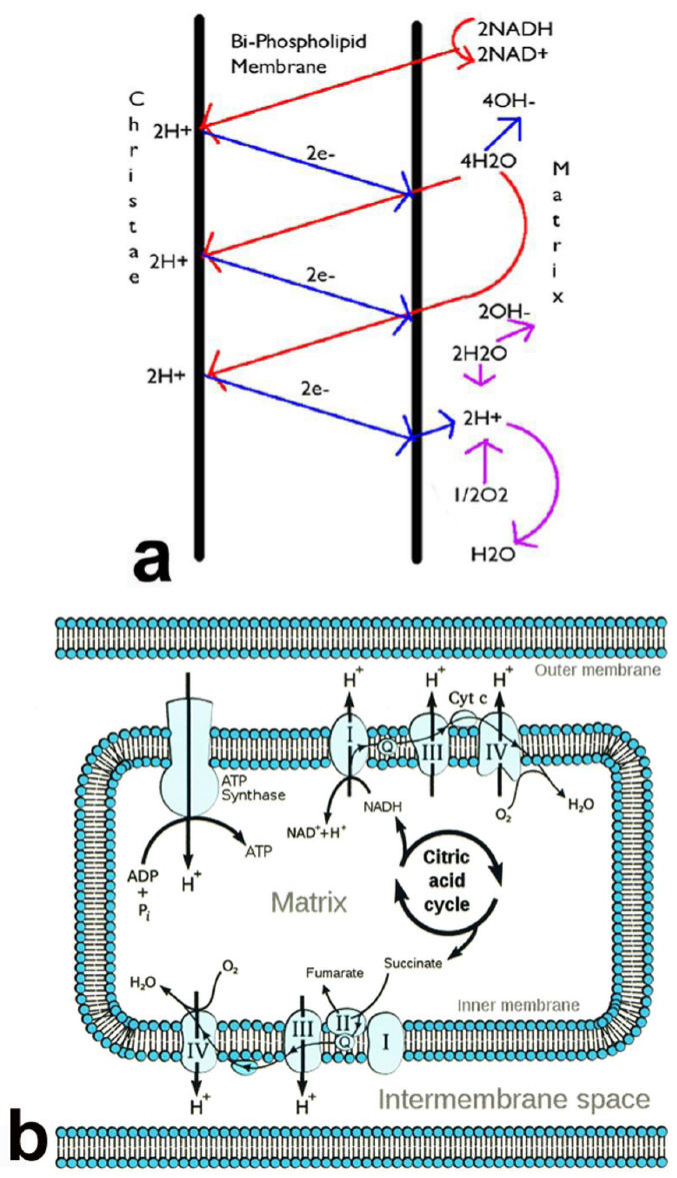
(**a**) Mitochondrial electrons combine with protons and molecular oxygen to produce water; (**b**) Mitochondria with enzymes involved in ATP energy synthesis depict relationship of outer membrane to the intermembrane space and inner membrane.

Organelle mitochondria of the cell produce more electrons and also acid during periods of lower oxygen concentrations [29,30], Figure 1. Subsequent increasing acid that provides growing hostile conditions with low pH in the biologic chlorine microenvironment adjacent to the metal implant can then create breakdown conditions of the generally corrosion-resistant passive TiO_2_ oxide layer to reinitiate more corrosion [22]. In addition to metabolic mitochondrial acid, the pH might become lower from inflammation and infection particularly if oxygen is blocked.

Different types of common corrosion have been classified for titanium implants. When acid breaks down the passive TiO_2_ oxide layer on a flat surface pitting corrosion occurs [1,2,21,22]. On the other hand, geometric implant material confinement of acid produces increased metal dissolution known at crevice corrosion [1,2,21,22]. Friction between the TiO_2_ oxide layer against another surface causes fretting corrosion [1,2,21,22]. When titanium is in direct contact with a dissimilar metal that is common to both oral and orthopedic implants galvanic corrosion occurs [1-3,21,22].

Subsequent electrochemical corrosion products from metal implants are thought to be damaging on local tissues particularly with respect to low intensity electromagnetic fields that are known to develop by corrosion and can then inhibit osteoblast growth [31]. Aseptic loosening of implants is thought to occur as a reaction to metal particles from corrosion that can produce an electric occurrence with electromagnetic field [31] where lower pH next to a titanium implant needs overall general consideration [20-28]. Titanium particles from implants are reduced in size by corrosion over time to commonly produce a dark blackened tissue stain [32]. Titanium particles found in adjacent soft tissue have been known to produce inflammation, fibrosis and necrotic tissue while infection was found to be a key reason for implant failure where pain was further noted as a clinical concern [33]. Microbial influences can also increase corrosion [1]. In terms of inflammation, titanium metal alloy particle release from implants can result in osteolysis or bone destruction [34]. Alternatively, after surgical implant placement chronic inflammation that continually heals can eventually form a fibrous capsule union between the implant and bone that leads to failure [35]. Also, inflammation appears to be increased at a disproportionate level to mechanical stress by a mineralized-type tooth/metal-implant interface solely as hard tissue connection without a normal fibrous tooth periodontal ligament [36] that forms a damping protective pad mechanism [15,16].

## 3. Infection

Implant failure on occasion is related to infection either directly even requiring antibiotic treatment or implant loosening from bone destruction with potential bacterial colonization [1,7]. Implant failure from infection is more injurious with greater complications and risks than aseptic failure [7,37]. The rate for infection with prosthetic hips ranges from approximately 0.2%–4% depending on the advanced level of surgery and hospital care [1,7] while infection with fewer less severe complications becomes more of a factor for dental implants that extend from bone into the oral cavity and other transcutaneous implants [7]. Infection can occur immediately related to implant surgical placement or years later by hematological transmission from a distant site through the blood from another location [1,7,37] or a break in the oral mucosa or skin. Further, implants increase the chance for bacterial infection by presenting a surface without a vascular blood supply and proper immune response [1]. Many bacteria are acidogenic/acidophillic to produce acid and also favor acidic growing conditions to metabolize complex organic compounds for a low pH capable of dissolving hydroxyapatite as enamel and dentin [38-40]. When acids lower the pH accelerated chemical degradation of polymer hydrocarbons and amines by hydrolysis occurs at increased rates [41-46] that could increase bacterial survival from organic nutritive breakdown products acquired through nearby tissue and cells. Also, cured epoxy polymer that contains different oxygen bonds [13] can be degraded *in situ* and *in vivo* [5].

Destructive low pH tissue environments next to metal implants build from metal Lewis acid corrosion products [21,23,24] while the implant surface prevents proper oxygen supply to cells for mitochondrial energy synthesis that produces both free radicals from the electron transport chain and acid from the proton gradient [26-30]. Subsequent rising acidic environments next to the implant add to chlorine surface interactions with titanium for increased corrosion [20-22,25]. Further, titanium metal is not known to integrate with soft tissue and form a seal that occurs with natural teeth to prevent oral bacterial contaminate leakage into bone. As a result, any disruptions in the implant/bone osseointegrated interface provide metal surface areas capable of allowing bacterial adhesion with mucopolysaccharide formation [1,47] and colonization for biofilm formation [1,7,37,47,48]. Also, implant surface roughness is a factor that improves bacterial adhesion [47]. In fact, implant properties that enhance osseointegration by protein adsorption also promote bacterial colonization [7]. Resulting implant biofilms protect bacteria colonies from host immune responses, antibiotics and allow bacteria to concentrate nutrients [1,7,37,47,48]. Implant biofilms even transmit along adjacent tissues to promote long-term infection [7]. In addition, bacterial colonization produces inflammatory responses that interfere with the bone/implant osseointegration [4]. Most implant infections do not show up in routine cultures because the biofilm protects bacterial colonies from releasing microbes [1]. However, because even small amounts of bacteria colonized can disrupt implant osseointegration, cases of aseptic loosening are being considered as subclinical bacterial contamination [7].

Loss of osseointegration through peri-implantitis as a destructive inflammation of bone supported by infection loosens tooth implants [6,36] with similar influences that are common to chronic adult periodontitis [36]. Numerous bacterial species identified from failed dental bone implants are analogous to those found with teeth in corresponding clinical conditions [36]. Frequently threaded implants for tooth/bone implants [36] might impose extra risk during progressive chronic implant bone loss by interfering with oral hygiene from difficult to clean inverted surfaces. Deeper titanium/bone implant infections do not have comparable conditions to clinical periodontitis where bone is resorbed distant from the periodontal pocket [36]. Because natural teeth have connections through perpendicular fibers of the periodontal ligament with bone while titanium implants produce parallel fibers that may not block bacterial penetration as well as teeth, remote bone loss may be a result in metal implants [36]. Staphylococci are the chief bacteria involved in orthopedic implant infections and can produce a biofilm after bacterial adhesion [1,37,47,49]. For later stage extraoral craniofacial implants, infections have most commonly been identified from skin bacterial species Staphylococcus aureus [4] that are becoming increasingly resistant to antibiotic treatment [4,37]. Other bacteria generally assist in chronic craniofacial implant infection with many different bacteria species identified [4].

## 4. Coatings

Titanium oxide surface layer forms instantly to a depth of 5–10 nm [3,22] in about a minute and continues to grow up to 200 nm as the reason for implant osseointegration with bone [49]. The most popular coating process using a plasma sprayed hydroxyapatite (HA) or Ca_10_(PO_4_)_6_(OH)_2_ produces a roughened surface texture that increases surface area to improve osseointegration bone attachment [3,49]. The mineral phase for bone is approximately 60% chiefly as HA with traces of other minerals and the remaining being 25% water and 15% organic compounds [1]. Increasing crystalline HA deposition slows coating release compared to lower HA crystalline deposition [3]. Commercial HA deposition ranges from 85% crystalline with 15% tricalcium phosphate or Ca_3_(PO_4_)_2_ to 97% crystalline [3]. However, controversy surrounds deposition of HA that shows improved bone growth next to the implant compared to the titanium metal surface but some studies suggest HA is detrimental over longer term use [3,49]. The bond for HA with metal is thought to be unstable and reduced following ion exchange over time with coating dissolution and even more dissolution of the tricalcium phosphate [3]. Increased failure of HA coatings over titanium metal is due to inflammation after coating dissolution and delamination [3] that would show as small defects to possibly protect bacteria hidden in safety for colonization. Loss of HA appears to reduce physiologic-type acid buffering by phosphate anion that is helpful under potential harsh lower pH conditions. Further, HA increases bacterial adhesion [3] while the HA roughened surface promotes bacterial adhesion growth [3,49] all of which contributes to peri-implantitis [3,49]. Also, modulus for HA osseointegration with adjacent bone is sufficiently rigid through less favorable energy dissipation to cause tissue reaction during applied stress at levels where pressure can also interfere with the HA coating durability [49]. Nitric acid passivates titanium [3] while electrochemical anodization is a relatively easy, inexpensive surface treatment used to increase surface texture and also improve the TiO_2_ surface with a thicker layer [3,35]. Defects in a metal crystal lattice scatter conduction electrons to increase resistivity [14]. Accordingly, the titanium oxide surface film produced at the anode has been shown to be less conductive with higher resistivity than the metal titanium [35,50] that may provide new biocompatibility properties for implant osseointegration [5,50]. In addition, the TiO_2_ surface thickness increases with increasing process temperatures that increases surface roughness, surface energy [50] and hardness [51] while reducing the contact angle [50] as a measure for increasing surface wetting [1]. No surface modifications have been found to counteract problems of infection other than uniform bone/implant osseointegration coverage contacts. Another area of interest that has shown possibilities for success include studies with bioactive bone morphogenic protein (BMP) in repairing bone defects to enhance bone growth next to the implant especially since proteins adsorb onto the implant surface before cell contact [49].

## 5. Polymer Matrix Composites (PMCs)

### 5.1. Results for PMC Biocompatibility

Osseointegration and antimicrobial properties are repeatedly hard to realize with titanium/titanium alloy implants [4], probably because biocompatibility with function is difficult using metal [52]. Although polymers have been identified for biomaterial use because of high biologic functionality, polymers lack mechanical strength needed with hard tissue implants [52]. In terms of polymer biocompatibility with sufficient strength, PMCs using high-strength fibers provide answers [5]. Fibers are the strongest and possibly the stiffest forms of a substance matter [53]. When combined into a thermoset cure crosslinking polymer matrix, fiber-reinforced composite materials provide design possibilities for ultimate potential in bone implant osseointegration toward biocompatibility with biofunction [5], Table 1. Most importantly, fiber-reinforced PMCs compete with metals especially on a strength-to-weight basis in required mechanical properties.

**Figure 2:**
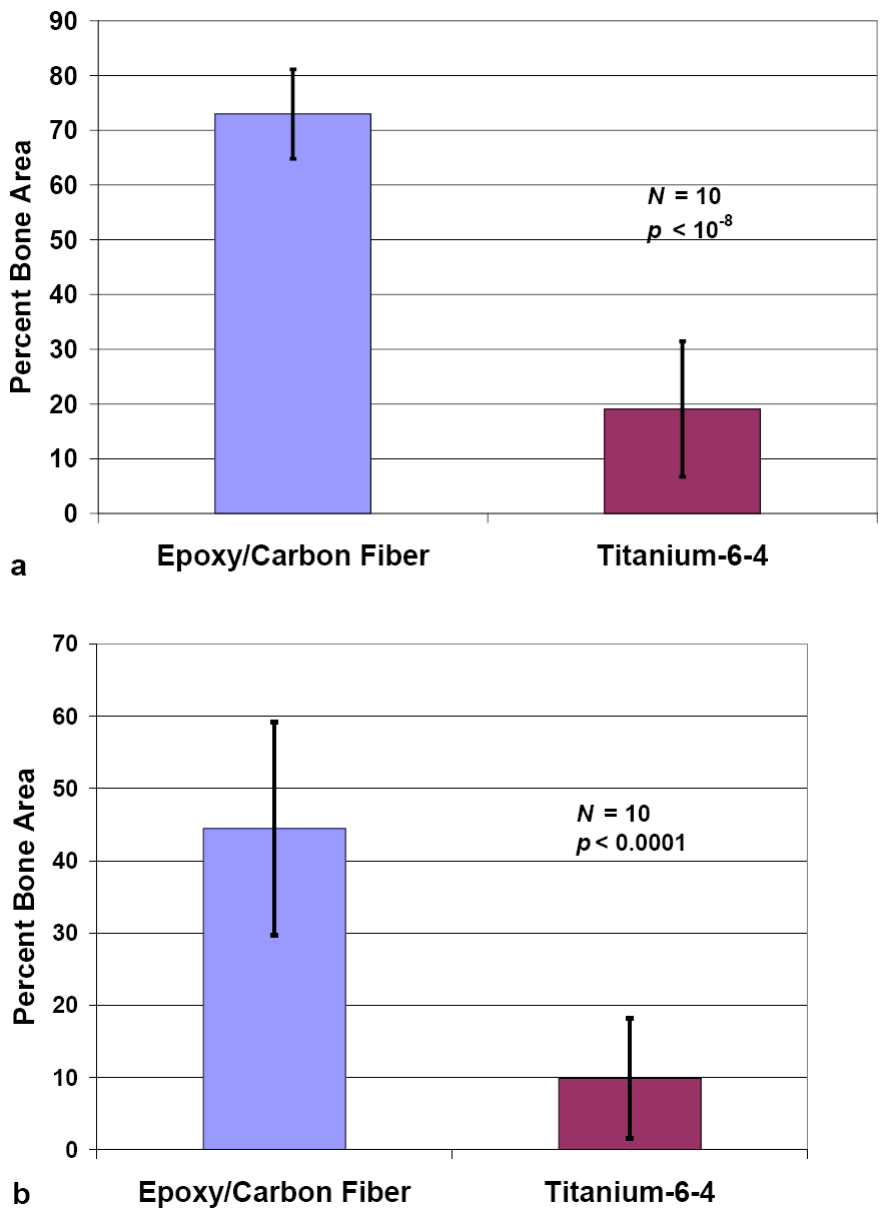
Implant percent bone areas comparing epoxy/carbon fiber PMC to Ti-6Al-4V alloy (**a**) Distance 0.1 mm from implant (**b**) Distance 0.8 mm from implant. (error bars ±1 standard deviation).

In comparison to a new bisphenol-epoxy/carbon fiber-reinforced composite implant material, titanium alloy Ti-6Al-4V produces significantly less bone forming near the implant with much lower levels of osseointegration contact in a bone-marrow animal implant model [5]. After two weeks, major breakthrough differences were apparent when comparing lateral cross-sectional percent bone area (PBA) for epoxy/carbon fiber PMC to Ti-6Al-4V alloy implanted midtibial *in vivo* using an animal model, Figure 2a,b [5]. At 0.1 mm distance from the implant PBA increased from 19.3 ± 12.3 with the titanium alloy implant to 77.7 ± 7.0 with the PMC, *p* < 10^−8^. At 0.8 mm distance PBA increased from 10.5 ± 5.3 with the metal alloy to 41.6 ± 13.9 with the PMC, *p* < 10^−4^ [5].

**Figure 3:**
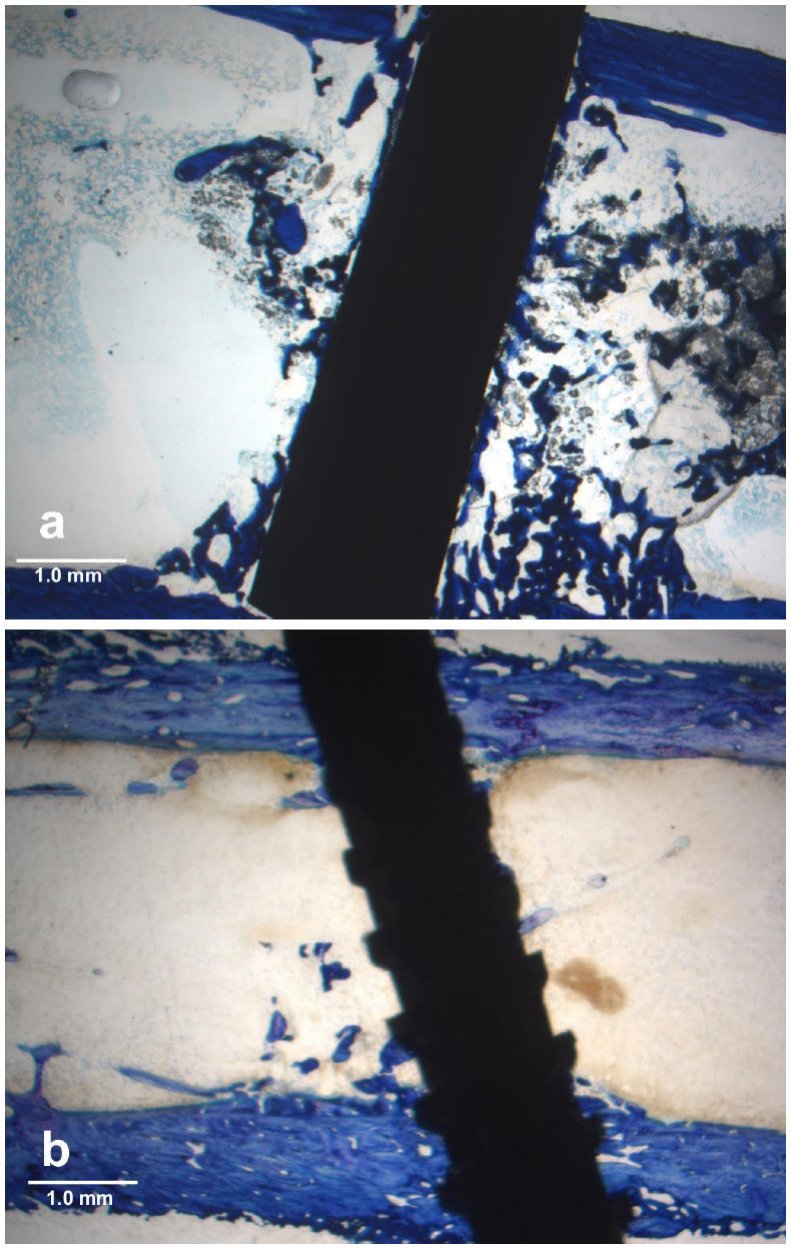
Lateral cross-sectional toluidine blue stain section at 2× magnification in a rat tibia bone marrow implant model (**a**) Epoxy carbon fiber PMC; (**b**) Titanium-6Al-4V alloy.

Typical histology ground sections for the epoxy carbon fiber PMC and titanium-6Al-4V alloy as average Histomorphometry PBA measurements are presented in Figure 3a,b.

**Figure 4:**
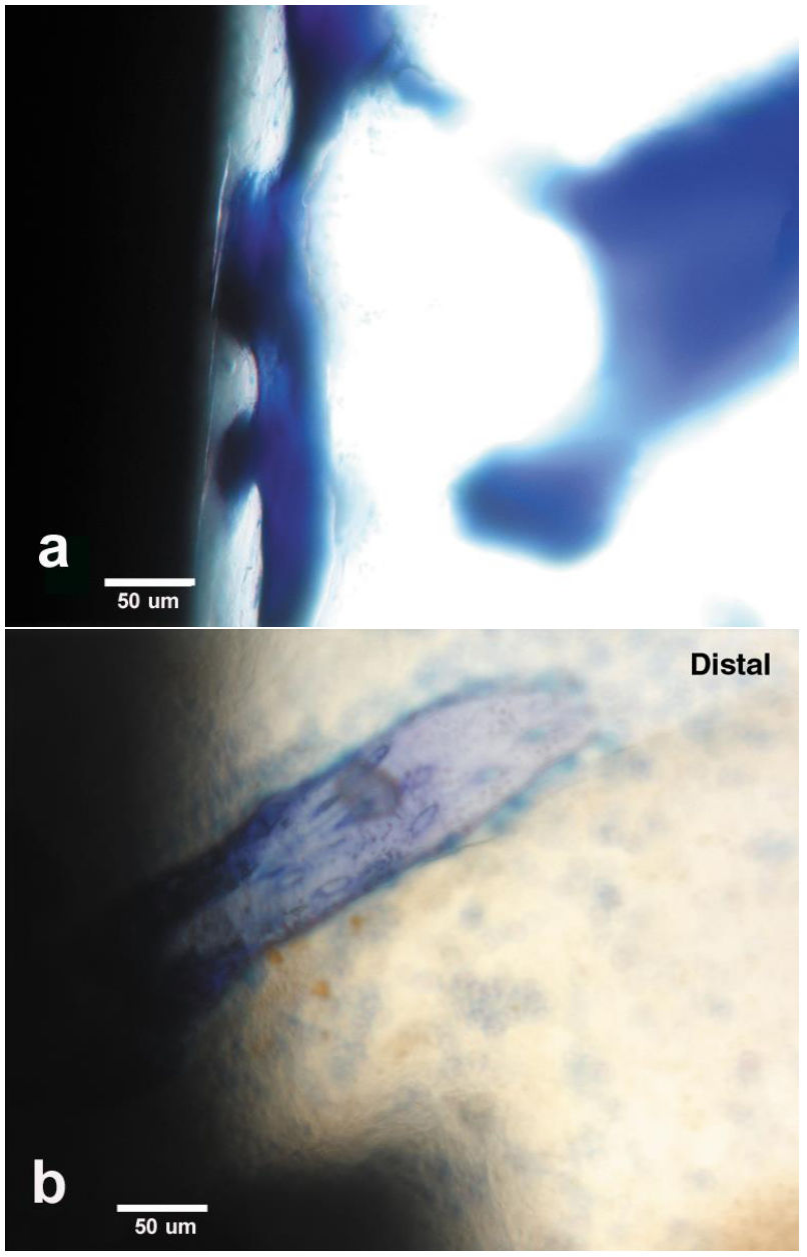
Toluidine blue stain osseointegration for lateral implant sections at 20× magnifications (**a**) Coordinated epoxy/carbon fiber osseointegration (**b**) Isolated titanium-6Al-4V osseointegration.

Osseointegration for the experimental epoxy carbon fiber PMC was broad along the length of the implant with structural pore-bearing organization for oxygen and nutrient accessibility. Conversely, titanium-6Al-4V alloy osseointegration was rare and nonstructured, Figure 4a,b.

**Figure 5:**
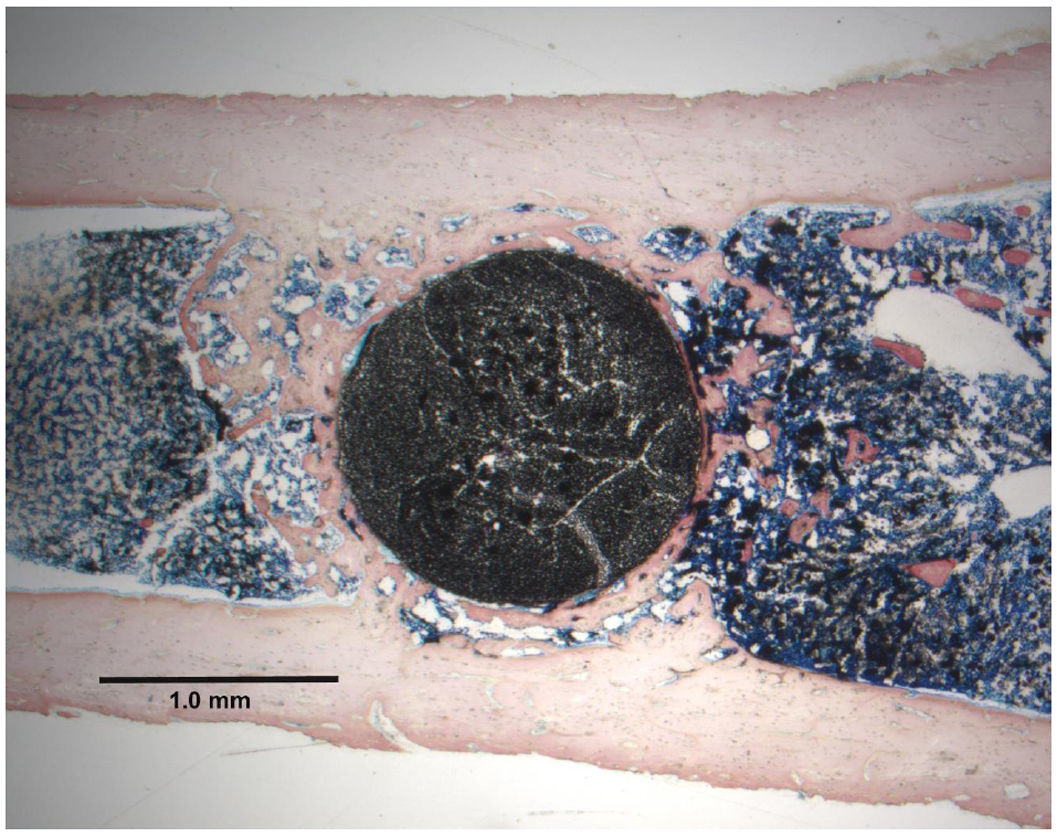
Sanderson’s stain epoxy/carbon fiber PMC horizontal section at 2× magnification in the marrow space shows mature organized pores in osseointegrating bone with the implant.

The extent of bone formation for the epoxy/carbon fiber PMC is presented with a horizontal section to better appreciate the exuberant extent of bone formation inside the bone marrow that is normally not seen physiologically, Figure 5.

**Figure 6:**
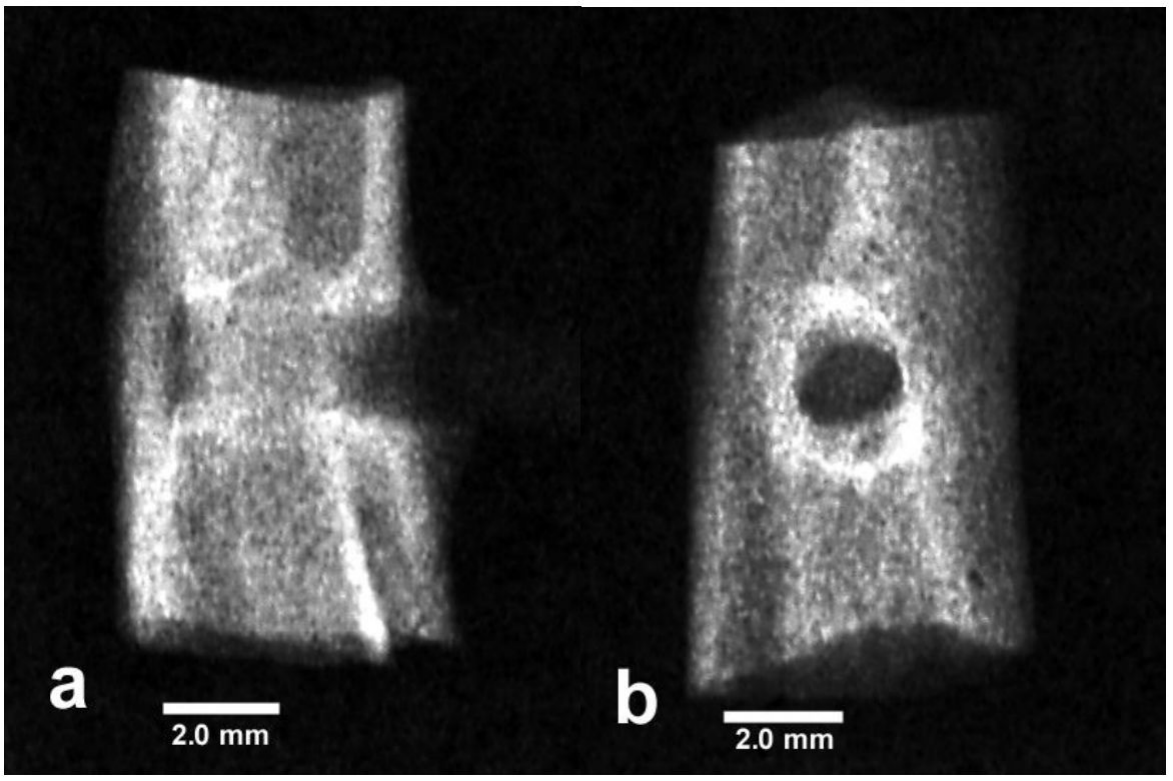
X-rays epoxy/carbon fiber PMC (**a**) Lateral view (**b**) Frontal view.

Normal difficult-to-see X-rays show how bone grows through the bone marrow space alongside the epoxy/carbon fiber PMC implant where bone does not usually grow, Figure 6a,b.

**Figure 7:**
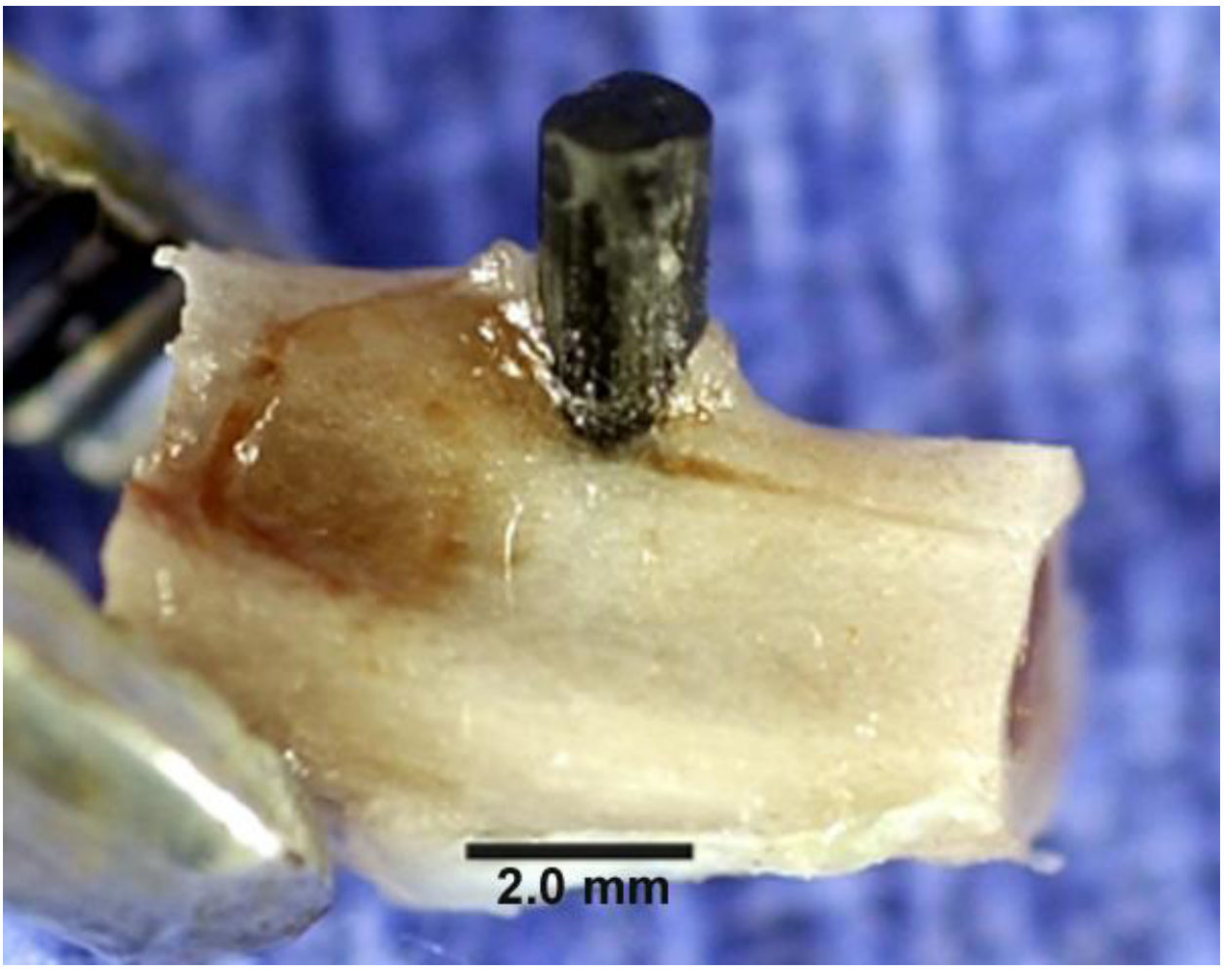
Photograph of epoxy/carbon fiber composite extending above tibial cortical bone with bone stimulated sufficiently to further grow upward along the side of the PMC carbon-fiber implant.

A photograph provides evidence of the strong osteogenic response for the epoxy/carbon fiber implant with bone growing above the outer cortical bone onto the PMC surfaces, Figure 7.

### 5.2. Nonpolar Molecular Attractions with Secondary Bonding

Bisphenol-epoxy/carbon-fiber PMC provides biocompatibility with biologic function through both the polymer matrix and fiber reinforcement [5]. Epoxy is a thermoset crosslinking cured polymer and considered polar or more accurately covalently polar in comparison particularly to nonpolar thermoplastic hydrocarbon-type polymers [13,24]. Because of the presence for possible retained amine, ether or epoxide groups with oxygen and nitrogen atoms in an epoxy polymer [13] increased polarity is expected for a nonpolar hydrocarbon [24]. A covalent bond is considered nonpolar when electrons are shared equally with electrons paired in overlapping orbitals [24]. However, when an electron pair is not shared equally when linking two atoms, the bond is considered covalent-polar at varying degrees depending on the nature of electron sharing connecting the two atoms involved [24]. The bond polarity is due to the electronegativity differences involving two separate atoms [24]. For example, bonds between two carbon atoms are identical and nonpolar while bonds with a carbon atom and hydrogen atom are basically nonpolar containing similar electronegativities for both the carbon and hydrogen atoms [24]. On the other hand, bonds linking carbon with oxygen or nitrogen are polar covalent with larger electronegativities for oxygen and nitrogen that more strongly attract the bonding pair of electrons [24]. Further, estrogen factors are present from bisphenol polymers [5,8,9-11,54] with a backbone derived from one of the first synthetic estrogens [5,8]. Subsequent physiologic actions of estrogen on bone include skeletal growth, increased osteoblast activity and retained Ca^2+^ and HPO_4_^2−^ mineralization due to organic bone matrix formation [30]. Also, estrogen and a precursor for resin, bisphenol A, protect the ovary from degeneration, uterine shrinking and bone loss in a concentration dependent manner [30,54]. Bisphenol A has also shown increased adult rat femur length without loss of strength [55] and decreased levels of micronuclei in bone marrow reticuloctyes [56]. In terms of biologic compatible uses, bisphenol A epoxy has approval level for food contact with coating the inside of food cans to resist corrosion [56] and in dental composite fillings [3,56].

For a biologic comparison, the cell membrane that comes in contact with a foreign implant material is composed of lipids, proteins and carbohydrates [30] all of which are similar in nature to polarity closer to the bisphenol epoxy than a metal. For instance, a cell membrane is approximately 50:50 lipid:protein by mass weight [30]. The membrane lipids are amphipathic with a hydrophilic (polar) globular head and hydrophobic (nonpolar) fatty acid tail [30]. Proteins as hydrocarbons with nitrogen and oxygen amide bonds are found inside the membrane and peripherally [30]. Cholesterol is a precursor to estrogen and found in the membrane to help maintain membrane fluidity [30]. Closed shell molecules attract one another through van der Waals forces because of the partial charges in polar covalent chemistry that further includes the small nonpolarity electronegative differences in hydrocarbons through multipolar effects [57] resulting in an intermesh of related molecular chains attracting one another. Subsequent similarities in molecular forces of attraction then exists in variation between the thermoset cure bisphenol polymers with the plasma cell membrane [5,30] and organic portions of the bone matrix [1,2] as forms of material biological function [5]. Consequently, bone-marrow precursor cells for the bone-forming osteoblasts apparently are recruited toward the bisphenol epoxy implant composite by similar chemical molecular structures to then form mature bone [5]. Regarding stress transfer, epoxy/carbon-fiber PMC bone plates have been compared with stainless steel and titanium in human forearm fractures to take advantage of lower modulus material with less stiffness and better bone response while most of the PMCs produced thin fibrous capsules grown next to the plates [58].

### 5.3. Carbon Fiber Biocompatibility

**Figure 8:**
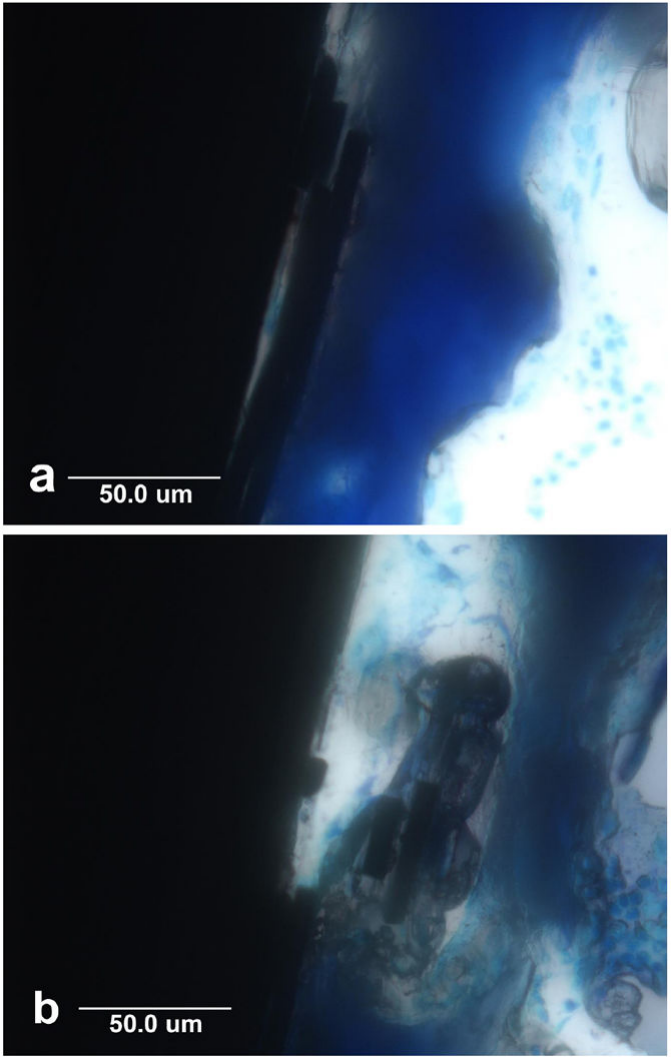
Lateral cross-sectional histology section at 40× magnification by toluidine blue of epoxy/carbon fiber PMC implant with broken carbon fibers pulled transversely away from the implant. (**a**) Carbon fibers are broken and pulled away from implant by bone in the transverse direction to open up small pore space at the PMC implant surface allowing minimal oxygen access; (**b**) After carbon fibers are split and pulled away from the PMC implant, bone osseointegrates entirely around small carbon fiber segments with a large pore remaining at the implant surface.

Carbon fibers also appear to stimulate strong cell recruitment during the extensive bone formation with the bisphenol epoxy implant PMC. Carbon fibers demonstrated extensive biocompatibility with bone as evidenced from the *in vivo* bone marrow implant testing through separate different mechanisms [5]. Carbon fibers are oxidized approximately 20% as received with R–COOH and R–COH surface groups [59] that should attract many biologic molecules similarly as hydrocarbons with oxygen through van der Waals forces [57]. Carbon fiber condensation reactions would provide strong covalent bonds through cell-membrane lipid fatty acids/phosphate/amino-acid end groups, bone phosphate and some organic portions of the bone matrix. Although fibers have strongest strengths in tension, fibers are weak in the transverse direction [14,53]. As a result, carbon fibers were found broken and in pieces alongside the implant with strong osseointegration bone association that could have pulled carbon fiber reinforcement sideways in the weak transverse direction, Figure 8a,b.

**Figure 9:**
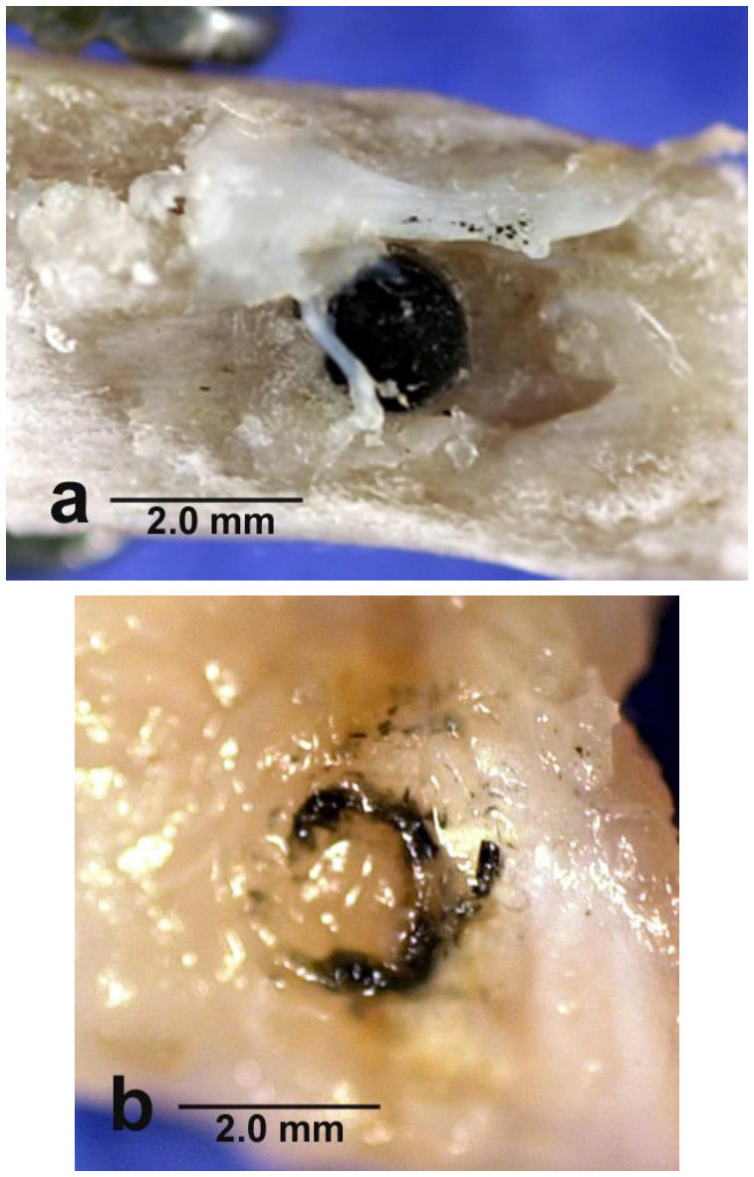
Photographs (**a**) implant extends above cortical bone with exuberant osteoid production stimulated from small carbon fiber fragments extruded out of the marrow space; (**b**) dissected soft tissue overlying the cortical bone integrated with carbon fiber fragments from the end of the implant.

Carbon fibers not only stimulate osteoid bone matrix formation, Figure 9a, but further encourage soft tissue attachments, Figure 9b. In fact, carbon fibers have been tested with apparent biocompatible success for ligament replacements in human knee reconstruction demonstrating concentric fibrous layers surrounding a carbon fiber core of mechanically sound intact fibers [60].

**Figure 10:**
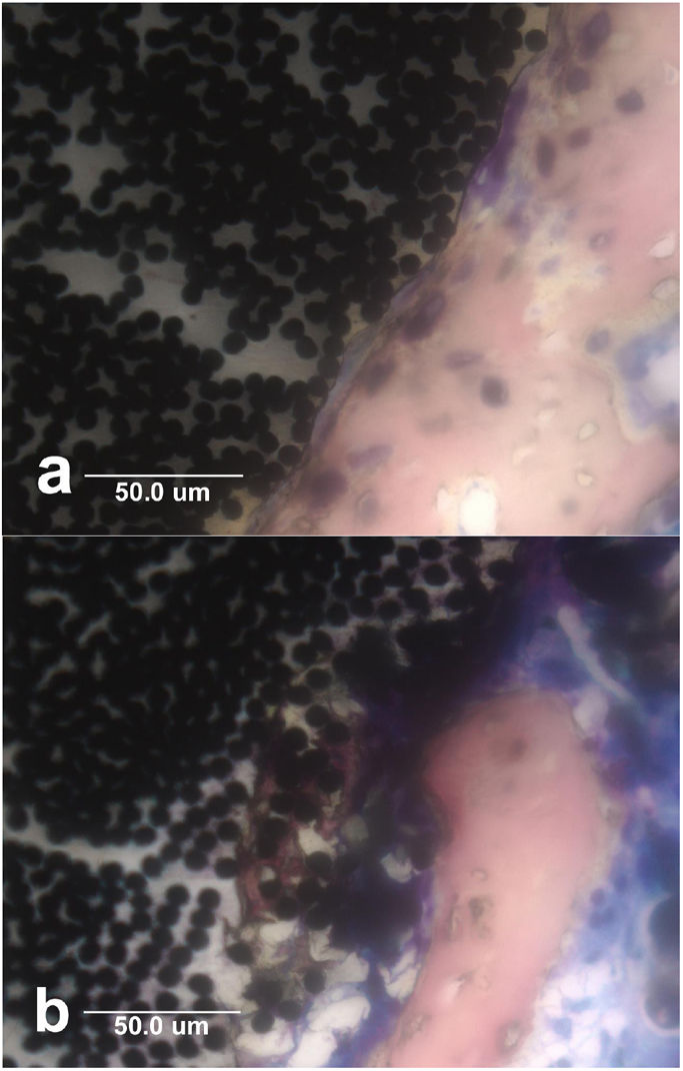
Horizontal cross-sectional histology with Sanderson’s stain at 40× magnification. (**a**) Bone osseointegration next to implant has softened the polymer matrix and pulled the surface outward into an irregular wave pattern and has also displaced carbon fibers; (**b**) Bone has osseointegrated with PMC inside an implant surface defect by degrading and replacing the polymer matrix with osteoid that has substantially surrounded individual carbon fibers approximately 50 μm into the implant.

Because normal low oxygen concentrations in bone marrow further produce acids during mitochondrial energy synthesis, epoxy polymer is softened and pulled away from the implant by bone attached with carbon fibers, Figure 10a. Small portions of carbon fiber are eventually degraded into a fine particulate smear layer on the very outer surface immediately next to the bone. Epoxy polymer is even broken down within the implant itself so that noncalcified osteoid is evident well into the implant and surrounding individual carbon fibers for heightened levels of osseointegration, Figure 10b.

By measure of bisphenol epoxy polymer degradation with depth of bone osseointegration into the carbon-fiber PMC, a defect in the implant surface can apparently reduce oxygen concentrations more than elsewhere to lower the pH. The osteocyte bone-forming cell involved tunnels into small spaces to extend cytoplasmic processes that secrete degrading enzymes and bone matrix proteins as osteoid [61]. Potential biologic relevant nitric acid chemistry has previously been considered in prior publications that it attacks bisphenol aromatic rings supported by a protein enzyme [5]. Figures showing bone to implant attachments indicate that covalent bonding with the carbon fibers by electron pair sharing is a chief bond mechanism for osseointegration while polymer covalent bonding appears possible. Also, mechanical retention develops as polymer degrades for strong bone ingrowth. On the other hand, titanium electron bonding is ionic with mineralization between bone and the TiO_2_ surface oxide layer.

Carbon fibers are electrically conductive [5,14] and with an insulating polymer coating become micro-biocircuits in a PMC [5]. Previous description of the implant microenvironment is found earlier with corrosion that describes the lower oxygen concentrations. As the distance increases from the blood supply oxygen concentrations become lower resulting in mitochondrial metabolic production of electrons and acid [5,26-30]. Subsequent mitochondrial electrons during hypoxia are then able to channel fast through carbon fibers electrochemically to areas of lower negative charge and lower electron concentrations [5]. Bone formed cells then preferentially seek carbon fibers to discharge excess electrons produced from the electron transport chain during mitochondrial energy synthesis concurrent with hypoxia, otherwise damaging free radicals could be produced [5]. Conductivity confers potential to remove inflammatory surgical free radicals [5] to form possible covalent bonds with exposed unpaired electrons [62] from the polymer by pH degradation. Overall, carbon fibers act as a permanent antioxidant to distribute free radicals that could prevent bone growth [5].

### 5.4. Electrical Biocompatibility and Semiconducting Properties

Electrical properties of cells have been studied most extensively at the plasma cell membrane level with a voltage potential of approximate −80 mV but can range from about −50 mV to −90 mV where the intracellular fluid is more negative with respect to the more positive extracellular biologic fluid [30]. The plasma cell membrane is composed of fluid lipid oils also structured intracellularly with protein fibers and extracellularly with divalent calcium that can form cements as calcium hydroxide and calcium oxide, form secondary bonds as calcium bicarbonate, produce inorganic mineral apatite as calcium phosphate, and thin elemental calcium channels [29]. Both protein fibers and fibrilar nanocalcium metals act as conducting biocircuits with small nanometer diameters to provide efficient electron flow [29]. Cell nanocircuits are important due to possible excessive electrons that need to be distributed through electrochemical gradients for uniformity to prevent high concentration build-ups that follow exponential rates for electron transfer [5,63]. However, unfortunate high electron current might be excessive and disintegrate small calcium or protein-type nanocircuits along the outside of the plasma cell membrane. However, semiconducting cellular materials that appear to exist at the plasma membrane phosphate-head-group/water interface next to susceptible extracellular nanocircuits [5,64] could safely adsorb and conduct excessive electrons until normal undamaging flow is reestablished. Similar use for semiconductors is well-known for microelectronic circuits that are stacked on top or lie within a silicon semiconductor wafer with a resistivity of approximately 3000 Ωm [14]. To better appreciate differences in electric currents that occur between metals that are conductors, polymer insulators and various semiconductors, resistivities are presented in Table 2.

In terms of potential problems arising without proper electron distribution, higher-than-normal electron concentrations can enter into free-radical crosslinking reactions to produce structured molecules [62]. The molecular structure then has the ability to interfere with normal biological diffusion or flow to prevent nutritive delivery to cells and even oxygen can be blocked that complicates physiology into pathological states [62,69]. Electron transfer reactions are extremely fast [63] and become particularly prevalent when free radical concentrations build which is the condition during disease with pathology [62,69] that should require fast conduction unloading of excess cell electrons. Also, high free-radical concentrations might encompass problems related to surgical inflammation as tissue heals. By similar free-radical electron transfer chemistry, biologic crosslinking could explain the coarse or clumping chromatin of DNA to DNA or DNA to protein [69] and protein agglomeration with insoluble accumulation [70,71] that overall could interfere with implant healing. Subsequent carbon-fiber-reinforced PMC has electrical conductivity/resistivity properties bordering on semiconducting bone properties also with polymer insulated carbon-fiber conductive biocircuits to support vital biocompatible physiological relationships [2,5,14,19] in preventing electron free-radical build up related to damaging increased molecular structure [62].

A safer semiconducting biomaterial surface will provide a more physiologic interface for better biocompatible faster electron transfer interaction with vulnerable nanocircuits of susceptible cell membranes [5]. As a well-studied relationship, titanium implant biocompatibility has been emphasized particularly with respect to the corrosion resistant surface titanium dioxide film. More specifically, TiO_2_ surface provides the special property for implant osseointegration with bone even at an extremely small thickness down in a range from 5–10 nm to 100–200 nm [49]. Resistivity values for titanium dioxide as a semiconductor are shown for mineralized rutile in a range of approximately 29–910 Ωm [65]. Corresponding similar relative semiconducting resistivity magnitudes are found with bone, a plasma cell membrane phospholipid/water interface model, physiologic saline and the new highly successful bisphenol-epoxy-polymer/carbon-fiber composite implant material, Table 2. Therefore, semiconduction apparently plays a role at some level in biocompatibility for implant osseointegration.

### 5.5. Stress Transfer

PMCs with carbon fiber reinforcement can supply densities/modulus much closer than titanium [5,14] to bone [1,2,5] for improved mechanical deformation by viscoelastic damping energy adsorption/dissipation [2,5,17] and healthy stress transfer with tissues/cell membranes [5]. Although carbon fibers appeared chemically inert, polymer softening by lowered pH created conditions that degraded the polymer with an expected much lower modulus for far easier deflection when mechanical stress was applied by bone.

### 5.6. Additives for Low Thermal PMC Processing

Thermal processing of epoxy PMC thermosets can range from room temperature cure up to less than 200 °C [13,53] compared to far higher temperatures for ceramics or metals [14]. Consequently, additives for epoxy PMC can include inorganic filler or organic compounds carefully selected for specific implant biocompatible design purposes. In addition to covalent bonding and polymer softening with bone ingrowth for osseointegration, ionic bonding mineralization by inorganic fillers as highly stable low-soluble crystalline HA can be provided. For cell recruiting, phosphate from HA could attract phosphate headgroups from phospholipid fatty acid plasma cell membranes and further calcium from HA could attract extracellular plasma cell membrane calcium that cements and mineralizes on the nanoscale for osseointegration potentials between forming bone and the implant. Also, phosphate anion acts as a physiologic-like buffer to counteract possible acids produced by hypoxia particularly next to an implant with inflammatory reactions from surgery. Particle HA can be infused as normal filler during the PMC resin infusion process with the carbon fibers. In fact, HA particle filler will be surrounded and retained by polymer completely so that the implant surface can be polished to a perfect smooth finish to reduce bacterial adhesion by most of the surface roughness mechanisms. In terms of preventing bacterial colonization, Triclosan is a highly stable hydrophobic and nonpolar crystalline powder antimicrobial that will incorporate into resin for PMC infusion [72].

### 5.7. Biocompatibility Coatings

Resorbable coatings are another feature to consider after the bulk material implant shape is set where thermal process can be controlled carefully for temperature sensitive proteins. Highly soluble calcium phosphate is an alternative to HA for rapid release during the stabilization phase with bone-to-implant osseointegration during healing. Tissue engineering design principles for bone implant osseointegration developed for long-term bulk-material application can then be applied for quick release in an outer resorbable coating to enhance quick implant stabilization with surrounding bone. As examples, low crystalline HA dissolves faster than highly stable crystalline HA to help speed initial bone growth, estrogen can enhance nonpolar lipid membrane and other organic attraction forces for improved cell recruitment, conductive particles or carbon nanotubes can draw in inflammatory free-radicals with other excess electrons and antimicrobial/antibiotics can be added to control bacteria introduced during surgical implant insertion.

## 6. Conclusions

Osseointegration bonding occurs by different covalent electron sharing and ionic mineralization mechanisms. TiO_2_ osseointegration produces ionic bonds by even oxidation states that act in coordination with the mineralization phase of bone. PMC osseointegration appears to produce covalent bonds by free-radical crosslinking with exposed unpaired electrons of the polymer following acid degradation while organic portions of the bone matrix or bone-cell plasma membrane condense by covalent bonding onto acid or hydroxyl groups of the oxidized carbon fibers. Further mechanical interlocking is achieved with rougher surfaces and with the PMC by acid degradation polymer removal can occur even with possible bone growth surrounding individual 7 μm diameter carbon fibers. Low pH polymer softening by acid is considered now to aid in adsorbing excessive stresses by a protective damping mechanism. Low temperature thermoset polymer cure allows fillers and organic additives to be incorporated by planned design with new tissue engineering for bone implants toward biosuccess. Fillers and additives can be included either in the bulk implant material that is polished to reduce microbial attachment colonization or in extremely mild resorbable coatings for rapid release to stabilize the initial implant surgical placement. Future research directions should examine implications clinically for the robust benefits and also surgical problems particularly during possible revision taking into account such strong osseointegration for the bisphenol-epoxy/carbon-fiber implant.

## Figures and Tables

**Table 1:** Biomaterial Properties

Material	Density (g/cm^3^)	Resistivity^a^ (Ω m)	Tensile Strength (MPa)	Yield Strength(MPa)	Modulus(GPa)
Bone longitudinal (Ω m radial-longitudinal 100% wet) [1, 2]	1.8-2.1	45-150	90-149	114	15.2-18.6
Titanium grades 1-4 [1, 2, 14]	4.5-4.51	10^-7^	240-550	170-485	104-110
Titanium-6-4aluminum vanadium alloy [1, 2, 14]	4.4-5.0	10^-8^	860-1103	795-1034	116-120
Bisphenyl Unidirectional CF^b^ [2, 14, 53, 54]	1.6	5	780-1850		140-325
Bisphenyl Unidirectional CF^b^ 4-pt. bend [2, 19, 29]	1.6	5	660-1800		64-255
Bisphenyl/CF^b^ Exp.Uni-woven laminate 4-pt. bend [19, 29]	1.49 (±.01)^c^	5	963 (±240)^c^	774 (±176)^c^	64 (±14.4)^c^
Bisphenyl 3-D Woven E-Glass 3-pt. Bend X-Y planes [19]			576 (±129)^c^	441 (±75)^c^	26 (±18)^c^
Unidirectional Photocure 3-pt Bend QF^b^ [29]			1118.8 (±207.6)^c^		76.6 (±13.3)^c^
Polymer Acrylic Bone Cement (PMMA) 4pt Bend [14, 29]	1.17-1.20	>10^12^	54.8 (±3.8)^c^	43.2 (±3.6)^c^	1.7 (±0.1)^c^

a Resistivity=1/Conductivity;

b CF (Carbon Fiber), QF (Quartz Fiber);

c Experimental standard deviations

**Table 2:** Resistivity^a^ of Different Engineering and Biological Materials

Material	Type	Resistivity (Ωm)
Titanium Pure	Conductor	4.2-5.2×10^-7^ [14]
Titanium-6Al-4V Alloy	Conductor	1.7×10^-8^ [14]
Titanium Dioxide (rutile)	Semiconductor	29-910 [65]
Bisphenol-Polymer/Carbon Fiber Composite	Semiconductor	5 [19]
Bone Longitudinal	Semiconductor	45-46 [2]
Bone Radial	Semiconductor	150 [2]
Physiologic Saline	Semiconductor	0.72 [2]
Silicon Pure	Semiconductor	3000 [66]
Silicon Phosphorous Doped	Semiconductor	20-80 [67]
Lipid Phosphate Headgroup/Water Interface	Semiconductor	100 [64]
Carbon Fibers	Conductor	9.5-18 × 10^-6^ [14]
General Metals	Conductors	~10^-6^-10^-9^ [14]
Thermoset Bisphenyl Epoxy Polymer	Insulator	10^10^-10^13^ [14]
Acrylic Bone Cement Polymer	Insulator	>10^12^ [14]
Pure Quartz Fiber	Insulator	10^20^ [68]

a Resistivity=1/Conductivity
